# Supplementary material for: Assessing the Environmental Drivers of Lassa Fever in West Africa: A Systematic Review
Source: Viruses. 2025 Mar 31;17(4):504. doi: 10.3390/v17040504 (PMC12031034; doi:10.3390/v17040504)
Supplement: Supplementary file 1 [file viruses-17-00504-s001.zip › viruses-3514456-supplementary.pdf]

Search strategy by database.

Google Scholar

Search number	Query	Date
1	Lassa virus OR fever AND environment	1/19/24
2	Lassa virus OR fever AND biodiversity	1/19/24
3	Lassa virus OR fever AND "biodiversity loss"	1/19/24
4	Lass virus OR fever AND land use	1/20/24
5	Lassa virus OR fever AND "land use change"	1/22/24
6	Lassa virus OR fever AND climate	1/22/24
7	Lassa virus OR fever and "climate change"	1/22/24
8	Lassa virus OR fever AND Mastomys natalensis OR Hylomyscus pamfi OR Mastomus erythroleucus	1/23/24
9	Lassa virus OR fever AND ecology	1/23/24
10	Lassa virus OR fever AND host	1/23/24
11	Lassa AND environment virus OR fever OR West OR Africa OR Sierra OR Leone OR Guinea OR Liberia OR Nigeria OR Côte OR "D Ivoire" OR Central OR Republic OR Mali OR Congo OR Senegal	1/23/24

12	Lassa AND biodiversity virus OR fever OR West OR Africa OR Sierra OR Leone OR Guinea OR Liberia OR Nigeria OR Côte OR "D Ivoire" OR Central OR Republic OR Mali OR Congo OR Senegal	1/23/24
13	Lassa virus OR fever OR "West Africa" OR Sierra OR Leone OR Guinea OR Liberia OR Nigeria OR Côte OR "D Ivoire" OR Central OR Republic OR Mali OR Congo OR Senegal "land use"	1/24/24
14	Lassa virus OR fever OR "West Africa" OR Sierra OR Leone OR Guinea OR Liberia OR Nigeria OR Côte OR "D Ivoire" OR Central OR Republic OR Mali OR Congo OR Senegal "land use change"	1/24/24
15	(Lassa AND (virus OR fever)) AND climate AND ("West Africa" OR Sierra OR Leone OR Guinea OR Liberia OR Nigeria OR Côte OR "D Ivoire" OR Central OR Republic OR Mali OR Congo OR Senegal)	1/24/24
16	(Lassa AND (virus OR fever)) AND "climate change" AND ("West Africa" OR Sierra OR Leone OR Guinea OR Liberia OR Nigeria OR Côte OR "D Ivoire" OR Central OR Republic OR Mali OR Congo OR Senegal)	1/24/24
17	(Lassa AND (virus OR fever)) AND "Mastomys	1/24/24

	natalensis" OR "Hylomyscus pamfi" OR "Mastomus erythroleucus" AND ("West Africa" OR Sierra OR Leone OR Guinea OR Liberia OR Nigeria OR Côte OR "D Ivoire" OR "Central Republic" OR Mali OR Congo OR Senegal)	
18	(Lassa AND (virus OR fever)) AND ecology AND ("West Africa" OR Sierra OR Leone OR Guinea OR Liberia OR Nigeria OR Côte OR "D Ivoire" OR "Central Republic" OR Mali OR Congo OR Senegal)	1/24/24
19	(Lassa AND (virus OR fever)) AND host AND ("West Africa" OR Sierra OR Leone OR Guinea OR Liberia OR Nigeria OR Côte OR "D Ivoire" OR "Central Republic" OR Mali OR Congo OR Senegal)	1/24/24

Pubmed search 1

Search number	Query	Date
1	("Lassa Fever"[Mesh]) OR "Lassa virus"[Mesh]	1/25/34
2	((((((((environment) OR (biodiversity)) OR (land use)) OR (climate)) OR (climate change)) OR (Mastomys natalensis)) OR (Hylomyscus pamfi)) OR (Mastomus	1/25/34

	erythroleucus)) OR (Ecology)) OR (Host*)	
3	(((((((((West Africa) OR (Sierra Leone)) OR (Guinea)) OR (Liberia)) OR (Nigeria)) OR (Côte D'Ivoir)) OR (Central African Republic)) OR (Mali)) OR (Congo)) OR (Senegal)	1/25/34
4	#1 AND #2 AND #3	1/25/34

Web of Science

Search number	Query	Date
1	("lassa fever" OR "lassa virus") AND (environment OR biodiversity OR "land use" OR climate OR "mastomys natalensis" OR "hylomscus pamfi" OR "mastomus erythroleucus" OR ecology OR host*) AND ("West Africa" OR "Sierra Leone" OR Guinea OR liberia OR nigeria OR ("Cote d'ivoir" OR "Ivory Coast") OR "Central African Republic" OR mali OR congo OR senegal)	2/2/24

BIOSIS

Search number	Query	Date
1	("lassa fever" OR "lassa virus") AND (environment OR biodiversity OR "land use" OR climate OR "mastomys natalensis" OR "hylomscus pamfi" OR	2/2/24

	"mastomus erythroleucus" OR ecology OR host*) AND ("West Africa" OR "Sierra Leone" OR Guinea OR liberia OR nigeria OR ("Cote d'ivoir" OR "Ivory Coast") OR "Central African Republic" OR mali OR congo OR senegal)	

Embase

Search number	Query	Date
1	("lassa fever" OR "lassa virus") AND (environment OR biodiversity OR "land use" OR climate OR "mastomys natalensis" OR "hylomscus pamfi" OR "mastomus erythroleucus" OR ecology OR host*) AND ("West Africa" OR "Sierra Leone" OR Guinea OR liberia OR nigeria OR ("Ivory Coast") OR "Central African Republic" OR mali OR congo OR senegal)	2/2/24

AJOL

Search number	Query	Date
1	("lassa fever" OR "lassa virus") AND (environment OR biodiversity OR "land use" OR climate OR "mastomys natalensis" OR "hylomscus pamfi" OR	2/2/24

	"mastomus erythroleucus" OR ecology OR host*) AND ("West Africa" OR "Sierra Leone" OR Guinea OR liberia OR nigeria OR ("Cote d'ivoir" OR "Ivory Coast") OR "Central African Republic" OR mali OR congo OR senegal)	

Pubmed search 2

Search number	Query	Date
1	("lassa fever" OR "lassa virus") AND (environment OR biodiversity OR "land use" OR climate OR "mastomys natalensis" OR "hylomscus pamfi" OR "mastomus erythroleucus" OR ecology OR host*) AND ("West Africa" OR "Sierra Leone" OR Guinea OR liberia OR nigeria OR ("Cote d'ivoir" OR "Ivory Coast") OR "Central African Republic" OR mali OR congo OR senegal)	2/2/24

Final set of included papers featured in Davis, et al.

Number	title	Year of Publiction	First Author
1	A mathematical model for Lassa fever transmission dynamics in a seasonal environment with a view to the 2017-20 epidemic in Nigeria	2021	Mahmoud A. Ibrahim
2	Lassa Fever 2016 Outbreak in Plateau State, Nigeria-The Changing Epidemiology and Clinical Presentation	2018	Nathan Y Shehu
3	Environmental-mechanistic modelling of the impact of global change on human zoonotic disease emergence: a case study of Lassa fever	2016	David W Redding
4	Lassa fever in Benin: description of the 2014 and 2016 epidemics and genetic characterization of a new Lassa virus	2020	Agnes Yadouleton
5	Hunting of peridomestic rodents and consumption of their meat as possible risk factors for rodent-to-human transmission of Lassa virus in the Republic of Guinea	1996	J Ter Meulen
6	Small mammal diversity and dynamics within Nigeria, with emphasis on reservoirs of the lassa virus	2018	Ayodeji Olayemi
7	Quantifying the seasonal drivers of transmission for Lassa fever in Nigeria.	2019	Andrei R Akhmetzhanov
8	Large-scale Lassa fever outbreaks in Nigeria: quantifying the association between disease reproduction number and local rainfall.	2020	Shi Zhao
9	Lassa fever in post-conflict sierra leone.	2014	Jeffrey G Shaffer
10	Infection pattern, case fatality rate and spread of Lassa virus in Nigeria.	2021	Clement Ameh Yaro
11	Descriptive epidemiology of Lassa fever in Nigeria, 2012-2017.	2020	Onyebuchi Augustin Okoro
12	Lassa Virus Circulation in Small Mammal Populations in Bo District, Sierra Leone.	2021	Umaru Bangura
13	Lassa virus isolation from Mastomys natalensis rodents during an epidemic in Sierra Leone	1974	T.P. Monath
14	Lassa virus infection in Mastomys natalensis in Sierra Leone. Gross and microscopic findings in infected and uninfected animals	1975	J.C. Demartini
15	Recent isolations of Lassa virus from Nigerian rodents	1975	Herta Wulff
16	A prospective study of the epidemiology and ecology of Lassa fever	1987	J.B. McCormick
17	Influence of landscape patterns on the exposure of LASV across diverse regions within the Republic of Guinea	2022	Stephanie Longet

18	Increased Prevalence of Lassa Fever Virus-Positive Rodents and Diversity of Infected Species Found during Human Lassa Fever Epidemics in Nigeria	2022	Anise N Happi
19	Predicting the evolution of the Lassa virus endemic area and population at risk over the next decades	2022	Raphaëlle Klitting
20	Circulation of Lassa virus across the endemic Edo-Ondo axis, Nigeria, with cross-species transmission between multimammate mice	2023	Adetunji Samuel Adesin
21	Modelling seasonality of Lassa fever incidences and vector dynamics in Nigeria	2023	James Q McKendrick
22	Spatio-temporal spread of Lassa virus and a new rodent host in the Mano River Union area, West Africa	2023	Umaru Bangura
23	Lassa virus in novel hosts: insights into the epidemiology of lassa virus infections in southern Nigeria	2024	Anise N Happi
24	Mastomys natalensis and Lassa fever, West Africa	2006	Emily Lecompte
25	Fluctuation of abundance and Lassa virus prevalence in Mastomys natalensis in Guinea, West Africa	2007	Elisabeth Fichet-Calvet
26	Reproductive characteristics of Mastomys natalensis and Lassa virus prevalence in Guinea, West Africa	2008	Elisabeth Fichet-Calvet
27	Prevalence and risk factors of lassa seropositivity in inhabitants of the Forest Region of Guinea: A cross-sectional study	2009	Solen Kernéis
28	Risk maps of lassa fever in West Africa	2009	Elisabeth Fichet-Calvet
29	Geographic Distribution and Genetic Characterization of Lassa Virus in Sub-Saharan Mali	2013	David Safronetz
30	The Impact of Human Conflict on the Genetics of Mastomys natalensis and Lassa Virus in West Africa	2012	Aude Lalis
31	Lassa serology in natural populations of rodents and horizontal transmission	2014	Elisabeth Fichet-Calvet
32	Sequence variability and geographic distribution of Lassa Virus, Sierra Leone	2015	Tomasz A Leski
33	The rubber plantation environment and Lassa fever epidemics in Liberia, 2008-2012: A spatial regression	2014	Babasola O Olugasa
34	Mapping the zoonotic niche of Lassa fever in Africa	2015	Adrian Q N Mylne
35	Host evolution in Mastomys natalensis (Rodentia: Muridae): An integrative approach using geometric morphometrics and genetics	2015	Aude Lalis

36	Spatial and temporal evolution of Lassa virus in the natural host population in Upper Guinea	2016	Elisabeth Fichet-Calvet
37	New Hosts of The Lassa Virus	2016	Ayodeji Olayemi
38	At home with mastomys and rattus: Human-rodent interactions and potential for primary transmission of lassa virus in domestic spaces	2017	Jesse Bonwitt
39	No measurable adverse effects of Lassa, Morogoro and Gairo arenaviruses on their rodent reservoir host in natural conditions	2017	Joachin Mariën
40	Prevalence of lassa virus among rodents trapped in three south-south states of Nigeria	2017	DE Agbonlahor
41	Movement Patterns of Small Rodents in Lassa Fever-Endemic Villages in Guinea	2018	Joachin Mariën
42	Widespread arenavirus occurrence and seroprevalence in small mammals, Nigeria	2018	Ayodeji Olayemi
43	Rodent control to fight Lassa fever: Evaluation and lessons learned from a 4-year study in Upper Guinea	2018	Almundena Mari Saez
44	Evaluation of rodent control to fight Lassa fever based on field data and mathematical modelling	2019	Joachin Mariën
45	Detection of antibody and antigen for Lassa virus nucleoprotein in monkeys from southern Nigeria	2019	Bamidele Nyemike Ogunro
46	Households as hotspots of Lassa fever? Assessing the spatial distribution of Lassa virus-infected rodents in rural villages of Guinea	2020	Joachin Mariën
47	Bayesian estimation of lassa virus epidemiological parameters: Implications for spillover prevention using wildlife vaccination	2020	Scott L Nuismer
48	An exploration of the protective effect of rodent species richness on the geographical expansion of lassa fever in West Africa	2021	Kyung-Duk Min
49	Bridging the gap: Using reservoir ecology and human serosurveys to estimate Lassa virus spillover in West Africa	2021	Andrew J Basinki
50	Detection of Lassa virus in wild rodent feces: Implications for Lassa fever burden within households in the endemic region of Faranah, Guinea	2021	Rebekah Wood
51	Domestic risk factors for increased rodent abundance in a Lassa fever endemic region of rural Upper Guinea	2021	Julia Clark
52	Geographical drivers and climate-linked dynamics of Lassa fever in Nigeria	2021	David W Redding

53	Two novel arenaviruses detected in pygmy mice, Ghana.	2013	Karl C Kronmann
54	Lassa fever in Guinea: II. Distribution and prevalence of Lassa virus infection in small mammals.	2001	Austin H Demby
55	Determining Ancestry between Rodent- and Human-Derived Virus Sequences in Endemic Foci: Towards a More Integral Molecular Epidemiology of Lassa Fever within West Africa.	2020	Ayodeji Olayemi
56	Detection of Lassa Virus, Mali	2010	David Safronetz
57	Lassa virus-infected rodents in refugee camps in Guinea: a looming threat to public health in a politically unstable region.	2007	Joseph Fair
58	Spatial distribution of commensal rodents in regions with high and low Lassa fever prevalence in Guinea	2005	Elisabeth Fichet-Calvet
59	A seasonal model to assess intervention strategies for preventing periodic recurrence of Lassa fever	2021	Saumen Barua
60	Mechanistic modelling of the large-scale Lassa fever epidemics in Nigeria from 2016 to 2019	2020	Salihu S Musa
61	Diversity and dynamics in a community of small mammals in coastal Guinea, West Africa	2009	Elisabeth Fichet-Calvet
62	Rodent-borne infections in rural Ghanaian farming communities	2019	Shirley C Nimo-Paintsil
63	a Gis-Based Approach to Risk Mapping of Lassa Fever Outbreak in Akure South Local Government Area, Nigeria	2022	Oluwafemi John Ifejube
64	The observed seasonal variation pattern and changing epidemiology of Lassa viral hemorrhagic fever disease in Ondo State, Nigeria	2020	Patrick Olanrewaju Osho
65	Ecological correlates and predictors of Lassa fever incidence in Ondo State, Nigeria 2017–2021: an emerging urban trend	2023	Simon Cadmus
66	The Role of Weather in the Spread of Lassa Fever in Parts of Northern Nigeria	2021	Joyce Imara Nchom
67	Mapping of Lassa Fever Epidemics in Owo, Ondo State, Nigeria, 2018-2020: A Descriptive and Categorical Analysis of Age, Gender and Seasonal Pattern	2021	NY Ohemeng-Parker, BO Olugasa…
68	Spatial analysis of confirmed Lassa fever cases in Edo State, Nigeria, 2008–2014	2019	Owoicho Samuel Amifofum

69	Rodent trapping studies as an overlooked information source for understanding endemic and novel zoonotic spillover	2023	David Simmons
70	Local disease-ecosystem-livelihood dynamics: reflections from comparative case studies in Africa	2017	Melissa Leach

Availability of data collection forms and study protocol

Data collection forms and the study protocol can be accessed upon request of the corresponding author.

PRISMA 2020 Checklist

Section and Topic	Item #	Checklist item	Location where item is reported
**TITLE**			
Title	1	Identify the report as a systematic review.	Page 1
**ABSTRACT**			
Abstract	2	See the PRISMA 2020 for Abstracts checklist.	Page 2
**INTRODUCTION**			
Rationale	3	Describe the rationale for the review in the context of existing knowledge.	Page 4
Objectives	4	Provide an explicit statement of the objective(s) or question(s) the review addresses.	Page 4-5
**METHODS**			
Eligibility criteria	5	Specify the inclusion and exclusion criteria for the review and how studies were grouped for the syntheses.	Page 5-6
Information sources	6	Specify all databases, registers, websites, organisations, reference lists and other sources searched or consulted to identify studies. Specify the date when each source was last searched or consulted.	Page 5
Search strategy	7	Present the full search strategies for all databases, registers and websites, including any filters and limits used.	Figure 1, Supplementary 1, Page 5-6
Selection process	8	Specify the methods used to decide whether a study met the inclusion criteria of the review, including how many reviewers screened each record and each report retrieved, whether they worked independently, and if applicable, details of automation tools used in the process.	Page 5-6
Data collection process	9	Specify the methods used to collect data from reports, including how many reviewers collected data from each report, whether they worked independently, any processes for obtaining or confirming data from study investigators, and if applicable, details of automation tools used in the process.	Page 6
Data items	10a	List and define all outcomes for which data were sought. Specify whether all results that were compatible with each outcome domain in each study were sought (e.g. for all measures, time points, analyses), and if not, the methods used to decide which results to collect.	Page 6
	10b	List and define all other variables for which data were sought (e.g. participant and intervention characteristics, funding sources). Describe any assumptions made about any missing or unclear information.	N/A
Study risk of bias assessment	11	Specify the methods used to assess risk of bias in the included studies, including details of the tool(s) used, how many reviewers assessed each study and whether they worked independently, and if applicable, details of automation tools used in the process.	Page 5
Effect measures	12	Specify for each outcome the effect measure(s) (e.g. risk ratio, mean difference) used in the synthesis or presentation of results.	N/A
Synthesis methods	13a	Describe the processes used to decide which studies were eligible for each synthesis (e.g. tabulating the study intervention characteristics and comparing against the planned groups for each synthesis (item #5)).	Page 6
	13b	Describe any methods required to prepare the data for presentation or synthesis, such as handling of missing summary statistics, or data conversions.	Figure 3, Page 9
	13c	Describe any methods used to tabulate or visually display results of individual studies and syntheses.	Figure 3, Page 9
	13d	Describe any methods used to synthesize results and provide a rationale for the choice(s). If meta-analysis was performed, describe the model(s), method(s) to identify the presence and extent of statistical heterogeneity, and software package(s) used.	Page 6
	13e	Describe any methods used to explore possible causes of heterogeneity among study results (e.g. subgroup analysis, meta-regression).	N/A
	13f	Describe any sensitivity analyses conducted to assess robustness of the synthesized results.	N/A
Reporting bias	14	Describe any methods used to assess risk of bias due to missing results in a synthesis (arising from reporting biases).	Page 6, 19

Section and Topic	Item #	Checklist item	Location where item is reported
assessment			
Certainty assessment	15	Describe any methods used to assess certainty (or confidence) in the body of evidence for an outcome.	Page 19
**RESULTS**			
Study selection	16a	Describe the results of the search and selection process, from the number of records identified in the search to the number of studies included in the review, ideally using a flow diagram.	Figure 1
	16b	Cite studies that might appear to meet the inclusion criteria, but which were excluded, and explain why they were excluded.	N/A, though can provide a list
Study characteristics	17	Cite each included study and present its characteristics.	Page 9-15
Risk of bias in studies	18	Present assessments of risk of bias for each included study.	Can provide on request
Results of individual studies	19	For all outcomes, present, for each study: (a) summary statistics for each group (where appropriate) and (b) an effect estimate and its precision (e.g. confidence/credible interval), ideally using structured tables or plots.	Page 7-8
Results of syntheses	20a	For each synthesis, briefly summarise the characteristics and risk of bias among contributing studies.	N/A. No syntheses conducted
	20b	Present results of all statistical syntheses conducted. If meta-analysis was done, present for each the summary estimate and its precision (e.g. confidence/credible interval) and measures of statistical heterogeneity. If comparing groups, describe the direction of the effect.	N/A
	20c	Present results of all investigations of possible causes of heterogeneity among study results.	N/A
	20d	Present results of all sensitivity analyses conducted to assess the robustness of the synthesized results.	N/A
Reporting biases	21	Present assessments of risk of bias due to missing results (arising from reporting biases) for each synthesis assessed.	N/A
Certainty of evidence	22	Present assessments of certainty (or confidence) in the body of evidence for each outcome assessed.	Page 19 statement on biased studies removed from inclusion
**DISCUSSION**			
Discussion	23a	Provide a general interpretation of the results in the context of other evidence.	Page 15-19
	23b	Discuss any limitations of the evidence included in the review.	Page 19
	23c	Discuss any limitations of the review processes used.	Page 19, Page 5
	23d	Discuss implications of the results for practice, policy, and future research.	Page 15-19
**OTHER INFORMATION**			
Registration and protocol	24a	Provide registration information for the review, including register name and registration number, or state that the review was not registered.	N/A
	24b	Indicate where the review protocol can be accessed, or state that a protocol was not prepared.	S2 Table

PRISMA 2020 Checklist

Section and Topic	Item #	Checklist item	Location where item is reported
	24c	Describe and explain any amendments to information provided at registration or in the protocol.	N/A
Support	25	Describe sources of financial or non-financial support for the review, and the role of the funders or sponsors in the review.	Page 20
Competing interests	26	Declare any competing interests of review authors.	N/A
Availability of data, code and other materials	27	Report which of the following are publicly available and where they can be found: template data collection forms; data extracted from included studies; data used for all analyses; analytic code; any other materials used in the review.	S2 Table

From: Page MJ, McKenzie JE, Bossuyt PM, Boutron I, Hoffmann TC, Mulrow CD, et al. The PRISMA 2020 statement: an updated guideline for reporting systematic reviews. BMJ 2021;372:n71. doi: 10.1136/bmj.n71.
This work is licensed under CC BY 4.0. To view a copy of this license, visit https://creativecommons.org/licenses/by/4.0/
